# Dual inhibition of PFKFB3 and VEGF normalizes tumor vasculature, reduces lactate production, and improves chemotherapy in glioblastoma: insights from protein expression profiling and MRI

**DOI:** 10.7150/thno.44427

**Published:** 2020-06-05

**Authors:** Junfeng Zhang, Wei Xue, Kai Xu, Liang Yi, Yu Guo, Tian Xie, Haipeng Tong, Bo Zhou, Shunan Wang, Qing Li, Heng Liu, Xiao Chen, Jingqin Fang, Weiguo Zhang

**Affiliations:** 1 Department of Radiology, Daping Hospital, Army Medical University, Chongqing, 400042, China.; 2 Department of Neurosurgery, Daping Hospital, Army Medical University, Chongqing, 400042, China.; 3 Department of Oncology, Daping Hospital, Army Medical University, Chongqing, 400042, China.; 4 Department of Radiology, PLA Rocket Force Characteristic Medical Center, Beijing, 100088, China.; 5 Department of Nuclear Medicine, Daping Hospital, Army Medical University, Chongqing, 400042, China.; 6 Chongqing Clinical Research Center of Imaging and Nuclear Medicine, Chongqing, 400042, China.

**Keywords:** glioblastoma, tumor vascular normalization, PFKFB3, bevacizumab, MRI

## Abstract

**Rationale:** Tumor vascular normalization (TVN) is emerging to enhance the efficacy of anticancer treatment in many cancers including glioblastoma (GBM). However, a common and severe challenge being currently faced is the transient TVN effect, hampering the sustained administration of anticancer therapy during TVN window. Additionally, the lack of non-contrast agent-based imaging biomarkers to monitor TVN process postpones the clinical translation of TVN strategy. In this study, we investigated whether dual inhibition of VEGF and the glycolytic activator PFKFB3 could reinforce the TVN effect in GBM. Dynamic contrast-enhanced-magnetic resonance imaging (DCE-MRI) and intravoxel incoherent motion (IVIM)-MRI were performed to monitor TVN process and to identify whether IVIM-MRI is a candidate or complementary imaging biomarker for monitoring TVN window without exogenous contrast agent administration.

**Methods:** Patient-derived orthotopic GBM xenografts in mice were established and treated with bevacizumab (BEV), 3PO (PFKFB3 inhibitor), BEV+3PO dual therapy, or saline. The vascular morphology, tumor hypoxia, and lactate level were evaluated before and at different time points after treatments. Doxorubicin was used to evaluate chemotherapeutic efficacy and drug delivery. Microarray of angiogenesis cytokines and western blotting were conducted to characterize post-treatment molecular profiling. TVN process was monitored by DCE- and IVIM-MRI. Correlation analysis of pathological indicators and MRI parameters was further analyzed.

**Results:** Dual therapy extended survival and delayed tumor growth over each therapy alone, concomitant with a decrease of cell proliferation and an increase of cell apoptosis. The dual therapy reinforces TVN effect, thereby alleviating tumor hypoxia, reducing lactate production, and improving the efficacy and delivery of doxorubicin. Mechanistically, several angiogenic cytokines and pathways were downregulated after dual therapy. Notably, dual therapy inhibited Tie1 expression, the key regulator of TVN, in both endothelial cells and tumor cells. DCE- and IVIM-MRI data showed that dual therapy induced a more homogenous and prominent TVN effect characterized by improved vascular function in tumor core and tumor rim. Correlation analysis revealed that IVIM-MRI parameter D^*^ had better correlations with TVN pathological indicators compared with the DCE-MRI parameter *K*^trans^.

**Conclusions:** Our results propose a rationale to overcome the current limitation of BEV monotherapy by integrating the synergistic effects of VEGF and PFKFB3 blockade to enhance chemotherapy efficacy through a sustained TVN effect. Moreover, we unveil IVIM-MRI parameter D^*^ has much potential as a complementary imaging biomarker to monitor TVN window more precisely without exogenous contrast agent injection.

## Introduction

Glioblastoma (GBM) is the most common and aggressive brain tumor in adults 1. Despite tremendous advances in emerging diagnostics and therapeutics, the prognosis of GBM patients is still dismal with a median survival time of 14.6 months 2. It has been recognized that the judicious use of antiangiogenic agents such as bevacizumab (BEV), which is a human recombinant vascular endothelial growth factor (VEGF) monoclonal antibody, normalizes tumor vasculature deriving a favorable prognosis in preclinical models and GBM patients 3-6. Moreover, tumor vascular normalization (TVN) offers a window of opportunity to improve chemotherapy effectiveness by remodeling a favorable tumor microenvironment and enhancing drug delivery 3,7,8. However, although TVN provides a promising paradigm for improving the efficacy of anticancer therapy, the window of opportunity is transient due to the short-lived normalization effect 9. Resistance to antiangiogenic therapy (AAT) develops rapidly in GBM thus the closure of normalization window 9-11. Therefore, it is an urgent need to develop novel strategies overcoming such therapeutic resistance to fortify TVN effect.

Tumor metabolic reprogramming confers AAT resistance 12,13. AAT induces metabolic adaptation shifting into anaerobic glycolysis in GBM 10,14,15. This adaptive mechanism stimulates the secretion of alternative proangiogenic cytokines and generates post-translational protein modification to compensate for the proangiogenic effect of VEGF pathway 14,16. Glycolysis is of importance in controlling angiogenesis 17. Endothelial cells (ECs) are addicted to glycolysis rather than mitochondrial respiration to generate localized adenosine triphosphate at lamellipodia and filopodia, thus increasing tip cell migration and stalk cell proliferation to form blood vessels 18. This glycolysis-driven process is mediated by the key glycolytic activator 6-phosphofructo-2-kinase/fructose-2, 6-bisphosphatase-3 (PFKFB3) 17. Previous studies have reported that PFKFB3 deficiency in ECs, or pharmacologically inhibiting PFKFB3 by 3-(3-Pyridinyl)-1-(4-pyridinyl)-2-propen-1-one (3PO), causes vascular defection and reduces pathological angiogenesis 17,19. Therefore, based on the role of PFKFB3 in angiogenesis and AAT resistance, we hypothesized that dual inhibition of PFKFB3 and VEGF could reinforce the TVN effect in GBM, thereby remodeling tumor microenvironment to create a more sustained window of opportunity to improve chemotherapy outcome.

Monitoring TVN dynamically is the critical prerequisite to guide anticancer therapy administration during the normalization window. As a typical exquisite technique to visualize lesions noninvasively, magnetic resonance imaging (MRI) plays an important role in the evaluation of tumor response after treatment 20-22. The quantitative parameter *K*^trans^ derived from dynamic contrast-enhanced-MRI (DCE-MRI) has been well-recognized to evaluate tumor vascular functionality and used as the recommended endpoint of the AAT response according to Quantitative Imaging Biomarkers Alliance (QIBA) 23. However, the residual of Gd-based contrast agents (GBCAs) in the brain is becoming a focused clinical concern 24,25, which may impede DCE-MRI being used frequently to monitor TVN window for clinical applications. Recently, intravoxel incoherent motion-MRI (IVIM-MRI) has gained much attention as an alternative imaging technique to measure capillary microperfusion without concern for the GBCAs deposition 26,27, making it possible to be used repetitively in a short duration for evaluating therapy response in patients with renal insufficiency or contraindications to GBCAs. Therefore, it is of important clinical implications to investigate whether IVIM-MRI could be a candidate or complementary imaging biomarker to DCE-MRI for monitoring the TVN effect. This outcome may accelerate the clinical translation of TVN strategy.

In this study, we conducted a dual therapy targeting VEGF and PFKFB3 to investigate whether dual therapy enhances TVN effect in orthotopic patient-derived xenograft (PDX) models of GBM, thereby generating a favorable tumor microenvironment by improving tumor hypoxia, drug delivery, and reducing lactate production to gain a better outcome of chemotherapy. Furthermore, we performed DCE-MRI and IVIM-MRI to monitor the process of TVN and analyzed the correlations between MRI parameters and TVN pathological indicators.

## Materials and Methods

### Tumor material and cell culture

Human GBM tissues were collected from Daping Hospital under protocols compliant with Human Research Ethics Committees of Daping Hospital, Army Medical University (Chongqing, China). Informed consent was obtained. Tumor tissues were disaggregated and cultured as described previously 28,29. Human umbilical vein endothelial cells (HUVECs) were purchased from the cell bank of Chinese Academy of Science (Shanghai, China) and cultured in endothelial cell growth medium (Sigma, 211-500).

### Animal model

GBM cells were implanted intracranially into the male NOD-SCID mice (6-8 weeks, ~25 g, purchased from the laboratory animal center of Daping Hospital) as described previously 30. Tumor volumes were monitored longitudinally according to the formula [width]^2^ × [length] × ^1^/_2_ based on *T*_2_-weighted MR images. All the procedures of the animals were conducted in compliance with the guidelines of National Institute of Health and our institutional Animal Use Subcommittee of Daping Hospital.

### Drug treatment and study design

**Figure 1:**
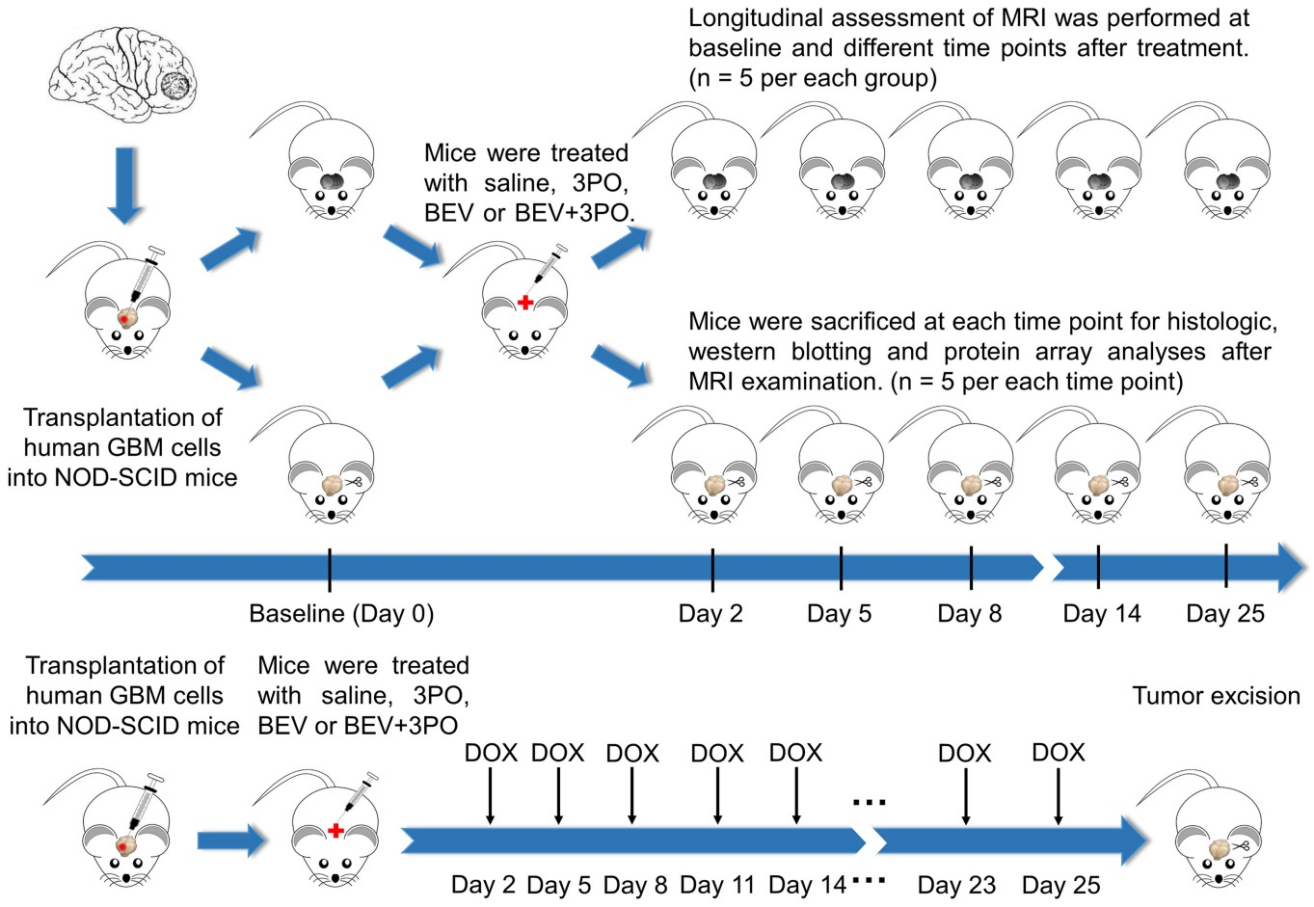
** Schematic of the study design.** Tumor-bearing mice were treated with different therapies and divided into MRI and histology subgroups. MRI and histology were conducted at different time points. For evaluation of drug delivery, DOX was administrated as indicated. Five mice in each group were sacrificed at day 25 for DOX accumulation evaluation.

After 14 days implantation, a total of 172 tumor-bearing mice were randomly divided into four groups (*n* = 43 per group) and treated either intraperitoneally with saline, BEV (5 mg/kg, biweekly; Roche), 3PO (25 mg/kg, three times a week; Sigma, 525330), or the combination of BEV and 3PO. Therapies were continued until the mice became moribund or displayed severe neurological symptoms (endpoint). The schematic of the study design was shown in Figure 1. Mice from each treatment group were randomized into the MRI subgroup (*n* = 5 per group) and histology subgroup (*n* = 30 per group), and then conducted longitudinal MRI scanning and histologic analysis at different time points, respectively. For survival study, mice (*n* = 8 per group) were monitored daily and killed humanely at the endpoint. For the evaluation of chemotherapeutic efficacy, 52 xenograft mice were used (*n* = 13 per group) and received intravenously doxorubicin (DOX; 2 mg/kg, three times a week; Sigma, D1515), DOX+3PO, DOX+BEV or DOX+BEV+3PO. To assess drug delivery, 5 mice in each treatment group were sacrificed 2 h after DOX administration at day 25. The remaining were used for survival study.

### Immunohistochemistry and immunofluorescence

Murine brains were fixed in 4% paraformaldehyde, embedded in paraffin, and sliced into 5 μm-sections. Tissue sections were deparaffinized and rehydrated followed by antigen retrieval with Tris-EDTA buffer (Abcam, ab93684). After blocking in TBS-Tween20 (TBST; Cell Signaling Technology, 9997) with 5% goat serum (Bioss, C-0005), the sections were incubated with the primary antibodies overnight at 4 °C. HRP-conjugated IgG secondary antibody (Cell Signaling Technology, 8114S) and 3, 3^'^-diaminobenzidine (DAKO) were used for the primary antibody detection. Alexa Fluor 488- (Beyotime Biotechnology, A0428) and 647- (Beyotime Biotechnology, A0468) conjugated secondary antibodies were used for immunofluorescence. Primary antibodies used included: Abcam: Ki67 (ab15580), collagen IV (ab6586), PFKFB3 (ab181861), CD31 (ab28364), α-smooth muscle actin (αSMA; ab7817), lactate dehydrogenase-A (LDHA; Cell Signaling Technology, 3582). To examine tumor hypoxia and cell apoptosis, pimonidazole (PIMO; Hypoxyprobe^TM^-1 Plus Kit, HPI Inc.) and TUNEL (Roche, 11684795910) staining were performed following the manufacturer's instructions. For the evaluation of DOX delivery, mice brains were harvested 2 h after DOX administration, snap-frozen in liquid nitrogen, and sliced into 10 μm-sections to observe. All sections were visualized and captured by confocal laser scanning microscopy (TCS SP8, Leica). Three typical fields per section were selected and analyzed using Image Pro-Plus 6.0 (Media Cybernetics). The “hot-spot” method was used for the quantification of microvascular density (MVD) 31. Pericyte coverage index (PCI) was defined as the ratio of positive αSMA to CD31 staining 32.

### Western blotting

Tumor tissues in RIPA buffer containing protease inhibitor (Servicebio, G2002) were homogenized, ice immersed, and vibrated for complete cell lysis. HUVECs and GBM cells treated with BEV (0.25 mg/mL) or BEV plus 3PO (0.20 μM) at 37 °C for 24 h were collected and lysed with RIPA buffer containing protease inhibitor. Proteins were separated by SDS/PAGE and transferred to PVDF membranes (Millipore, IPVH00010). Membranes were incubated with anti-PFKFB3 (Abcam, ab181861), anti-Tie1 (Abcam, ab111547), and anti-β-actin (Servicebio, GB11001) in blocking buffer (5% skimmed milk in TBST) at 4 °C overnight. HRP-conjugated secondary antibodies were used for detection. The blots were developed using enhanced chemiluminescence reagent (Servicebio, G2014) and then quantified with AlphaEase FC software (Alpha Innotech).

### Angiogenesis cytokine array

Tumor tissues were collected at day 25 after treatments to perform human cytokine antibody microarrays (QAH-ANG-2/QAH-ANG-3, RayBiotech) according to the manufacturer's instructions. The data were analyzed by the R package (Bioconductor). Protein expression with a minimum fold change of 1.2 and a maximum adjusted *P*-value of 0.05 was deemed as differentially expressed.

### MRI and data postprocessing

MRI experiments were performed using a 7T Pharmascan (Biospin70/20, Bruker) equipped with a four-channel mouse head transmitter/receiver coil. Scanning sequences included as follow: *T*_2_-weighted images (Turbo-RARE, echo time (TE)/repetition time (TR): 45 ms/4000 ms, field of view: 25 × 25 mm, matrix: 256 × 256, slice thickness: 0.5 mm), IVIM-MRI (SE-EPI, TE/TR: 21 ms/2500 ms, field of view: 30 × 30 mm, matrix: 128 × 128, 12 *b* values: 0, 20, 40, 60, 80, 100, 120, 160, 200, 400, 800, and 1200 s/mm^2^, slice thickness: 0.5 mm), Magnetic resonance spectroscopy (MRS; PRESS, TE/TR: 16 ms/2500 ms, a single 2 × 2 × 2 mm^3^ voxel was located in the homogeneous tumor region based on *T*_2_-weighted images), *T*_1_ maps (FLASH, TE/TR: 2.8 ms/16.3 ms, field of view: 25 × 25 mm, matrix: 128 × 128, 6 flip angles: 5°, 10°, 15°, 20°, 25°, and 30°, slice thickness: 1 mm), DCE-MRI (same geometry as *T*_1_ maps, TE/TR: 2.78 ms/16.28 ms, flip angle: 25°) with gadodiamide (OMNISCAN, GE Healthcare) administrated intravenously (0.1 mM/kg of body weight). DCE-MRI images were processed by Omni-Kinetics software 2.0 (GE Healthcare) and the Tofts-Kermode model was used for the calculation of the quantitative parameter *K*^trans^. IVIM images were analyzed by nordicICE software 4.1.3 (NordicNeuroLab) to calculate the pseudo-diffusion coefficient (D^*^), perfusion fraction (*f*), and diffusion coefficient (*D*) using the biexponential model 27. Regions of interest (ROIs) of the whole tumor were drawn manually by delineating the tumor border on the corresponding parametric images showing the maximum cross-sectional tumor area 33. ROIs of two tumor subregions (tumor core and tumor rim) were defined as previously described 34,35. MRS data were analyzed using jMRUI software 5.2 for assessment of the relative levels of choline (Cho), N-acetyl aspartic acid (NAA), lactate (Lac), or lipid (Lip) to creatine (Cr).

### Statistics analysis

All numeric data were presented as the mean ± *SD* unless otherwise noted. The normal distribution of acquired data was assessed by the Kolmogorov-Smirnov test. The Student's *t*-test was used in the two-group comparison. One-way ANOVA was used for multiple comparisons among different groups. Pearson correlation was used to analyze the correlations between MRI parameters and histologic markers.* P* < 0.05 was considered statistically significant. All statistical analyses were performed using SPSS 19.0 (SPSS Inc.) and GraphPad Prism 7.0 (GraphPad Software Inc.).

## Results

### Dual therapy enhances the antitumor effect and extends survival compared with BEV monotherapy

**Figure 2:**
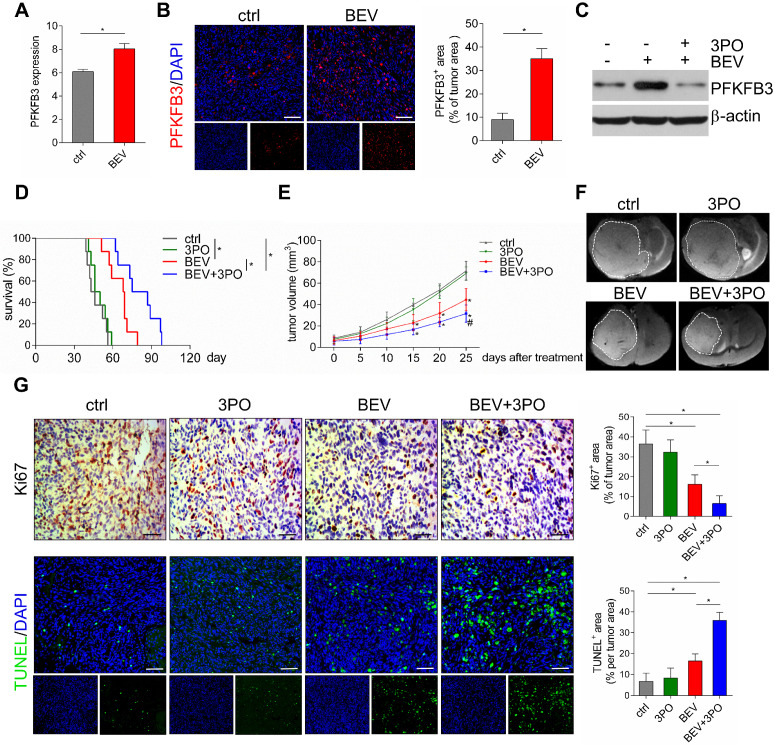
** The therapeutic effects of different treatments on survival, tumor growth, and cell proliferation and apoptosis. (A)** GEO profile of PFKFB3 expression in U87MG GBM models in control and BEV groups (*n* = 6). Representative images of PFKFB3-stained sections and quantification of PFKFB3^+^ areas in control and BEV-treated tumors at day 25 after treatments (*n* = 10). **(B)** Representative images of PFKFB3-stained sections and quantification of PFKFB3^+^ areas in control and BEV-treated tumors at day 25 after treatments (*n* = 10). **(C)** Western blotting of PFKFB3 in control, BEV, and BEV+3PO-treated tumors at day 25 after treatment (*n* = 9). **(D)** Survivals of tumor-bearing mice from different therapy groups (*n* = 32). **(E)** Growths of GBM tumors from different therapy groups (*n* = 20). **(F)** Representative *T*_2_-weighted images of tumor volumes from different therapy groups at day 25 after treatments (*n* = 20).** (G)** Representative Ki67- and TUNEL-stained sections and corresponding quantification from different therapy groups at day 25 after treatments (*n* = 20). Scale bars, 50 µm **(A, G)**. All data are means ± *SD*. ^*^P < 0.05, compared with control. ^#^*P* < 0.05 compared with BEV group.

Previous studies have demonstrated that anaerobic glycolysis is activated in GBM after BEV treatment 10,14. We hypothesized that this metabolic reprogramming is accompanied by elevated expression of the glycolytic activator PFKFB3. As expected, GEO data (GSE39221) revealed that PFKFB3 was highly expressed after BEV treatment in U87 GBM tissues (Figure 2A). This result was further confirmed in PDX GBM samples (Figure 2B). Moreover, we found that 3PO effectively inhibited BEV-induced PFKFB3 expression (Figure 2C). Based on the critical role of PFKFB3 in angiogenesis 17,19, a question was raised that whether inhibiting PFKFB3 by 3PO could improve the efficacy of BEV monotherapy. Therefore, we first investigated the impact of dual inhibition of VEGF and PFKFB3 on survival and tumor growth. Both BEV monotherapy and dual therapy significantly extended the survival of tumor-bearing mice compared with control. Strikingly, after 10 weeks of treatment, only the mice given dual therapy were still alive (Figure 2D and Table S1).

Given the improvement of survival in dual therapy over BEV monotherapy, we next examined the effect of dual therapy on tumor growth. The mean of tumor volume in BEV-treated mice and that in mice given BEV and 3PO were smaller than in control (Figure 2E and Figure S1). Notably, the growths of tumors treated with dual therapy delayed more significantly than that treated with BEV monotherapy at day 25 (Figure 2F). Cell proliferation and apoptosis evaluated by Ki67 and TUNEL staining further demonstrated that dual therapy reduced GBM cell proliferation and facilitated apoptosis more remarkably than BEV monotherapy does (Figure 2G).

### Dual therapy reinforces tumor vascular normalization compared with BEV monotherapy

**Figure 3:**
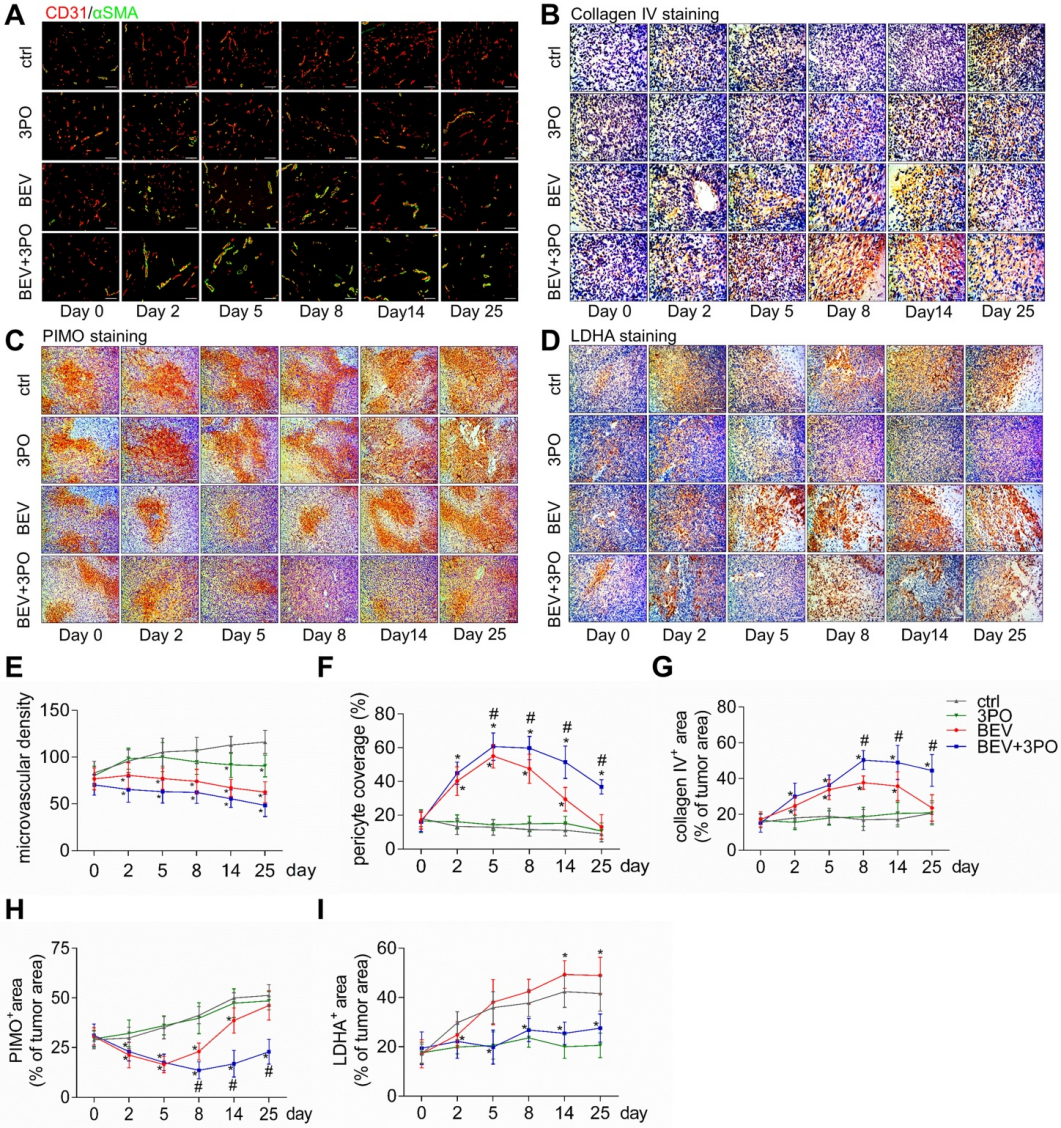
** Histological analysis of tumors at different time points after treatments.** Representative images of double CD31 and αSMA-stained **(A)**, collagenⅣ-stained **(B),** PIMO-stained **(C)**, and LDHA-stained **(D)** GBM sections at different time points from different treatment groups (*n* = 120). The longitudinal assessments of MVD **(E)**, pericyte coverage **(F)**, collagenⅣ expression **(G)**, tumor hypoxia **(H)**, and LDHA expression **(I)** in the control, 3PO, BEV, and BEV+3PO groups at different time points. Scale bars, 50 µm **(A, B)**, 100 µm **(C, D)**. All data are means ± *SD*. ^*^P < 0.05, compared with control. ^#^*P* < 0.05, compared with the BEV group.

Previous studies have demonstrated TVN is associated with increased survival and antitumor effect in preclinical models and GBM patients 4-6,36. Therefore, we hypothesized that the improved survival and antitumor efficacy by dual therapy may benefit from a more durable TVN effect. We conducted a longitudinal assessment of vascular morphology based on MVD and normalized features (PCI and collagen IV) 37. Tumor vessels were pruned gradually in all treatment groups with a greater extent in tumors treated with BEV or dual therapy (Figures 3A and 3E). PCI and collagen IV expression were dramatically higher after BEV or dual therapy with a greater extent in the latter, indicating that inhibiting PFKFB3 by 3PO enhances the normalization extent induced by BEV monotherapy. Moreover, dual therapy induced a longer TVN effect than that seen in tumors treated with BEV monotherapy. At day 8, PCI and collagen IV expression in BEV-treated tumors began to slump and further declined close to the baseline and control levels at day 25, suggesting the endpoint of TVN window. However, although the slight downtrends of PCI and collagen IV started at day 14, high PCI and collagen IV expressions in dual therapy-treated tumors were still maintained by day 25 (Figures 3A-3B and 3F-3G).

### Dual therapy alleviates tumor hypoxia and reduces LDHA and lactate levels

Given that tumor oxygenation status, as the central indicator of TVN, is improved during normalization window 3,38, we investigated whether these vascular changes could translate to alleviate tumor hypoxia. Using the hypoxia-probe by PIMO staining, we found that tumor hypoxia mitigated at day 5 in mice given BEV monotherapy but increased again at day 8. This uptrend reached a peak at day 25. In contrast, tumor hypoxia was improved as early as day 2 and maintained at a low level by day 25 in mice given BEV and 3PO (Figures 3C and 3H).

**Figure 4:**
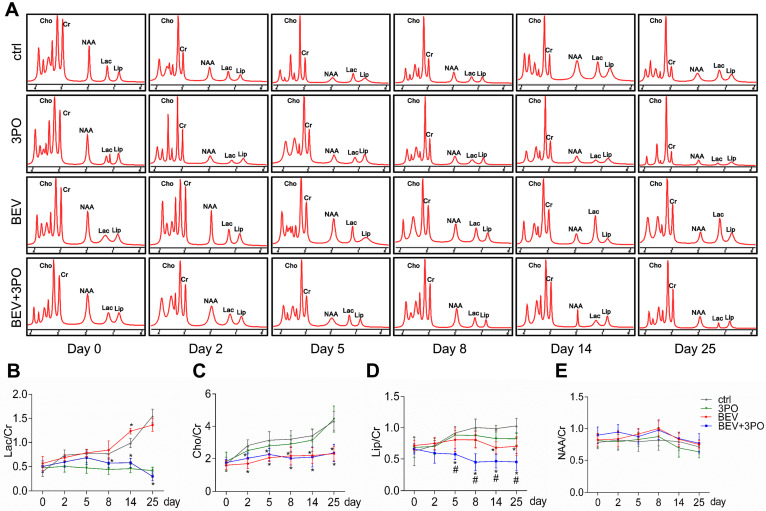
** MRS analysis of tumors at different time points after treatments. (A)** Representative images of MRS at different time points from different treatment groups (*n* = 20). The longitudinal assessments of Lac/Cr **(B)**, Cho/Cr **(C)**, Lip/Cr **(D)**, and NAA/Cr in the control, 3PO, BEV, and BEV+3PO groups at different time points. All data are means ± *SD*. ^*^P < 0.05, compared with control. ^#^*P* < 0.05, compared with the BEV group.

Having established that BEV treatment induces activated glycolysis in GBM characterized by increased LDHA expression and lactate production 10,39; we next investigated whether the addition of 3PO to BEV monotherapy enables the restrain of LDHA and lactate levels in GBM. Consistent with the previous study 14, LDHA expression in BEV-treated tumors was increased gradually and even more remarkable than tumors in control at day 14 and day 25. However, the LDHA expression in dual therapy lowered significantly, suggesting 3PO inhibits the BEV-induced LDHA expression (Figures 3D and 3I). We further studied the levels of lactate and other tumor metabolites by MRS. In line with the result of LDHA expression, a gradual rise of Lac/Cr level was observed in BEV-treated tumors, which was more prominent than tumors in control at day 14, indicative of the activation of tumor glycolysis and the deteriorate of acidic microenvironment after BEV treatment. Conversely, the addition of 3PO rendered BEV-induced Lac/Cr value to decline after 5-day treatment and reached the lowest value at day 25 (Figures 4A-4B). Additionally, although there is no difference of NAA/Cr among all groups, significant reductions of Cho/Cr and Lip/Cr were observed in BEV or dual therapy group with greater extent of Lip/Cr in the latter (Figures 4C-4E), suggesting a profound reduction of tumor cell proliferation and necrosis after dual therapy. Collectively, we found that BEV monotherapy leads to a more acidic tumor microenvironment characterized by increased LDHA and lactate levels. This effect is conversely neutralized when combined with 3PO.

### Dual therapy improves the efficacy of chemotherapy compared with BEV monotherapy

**Figure 5:**
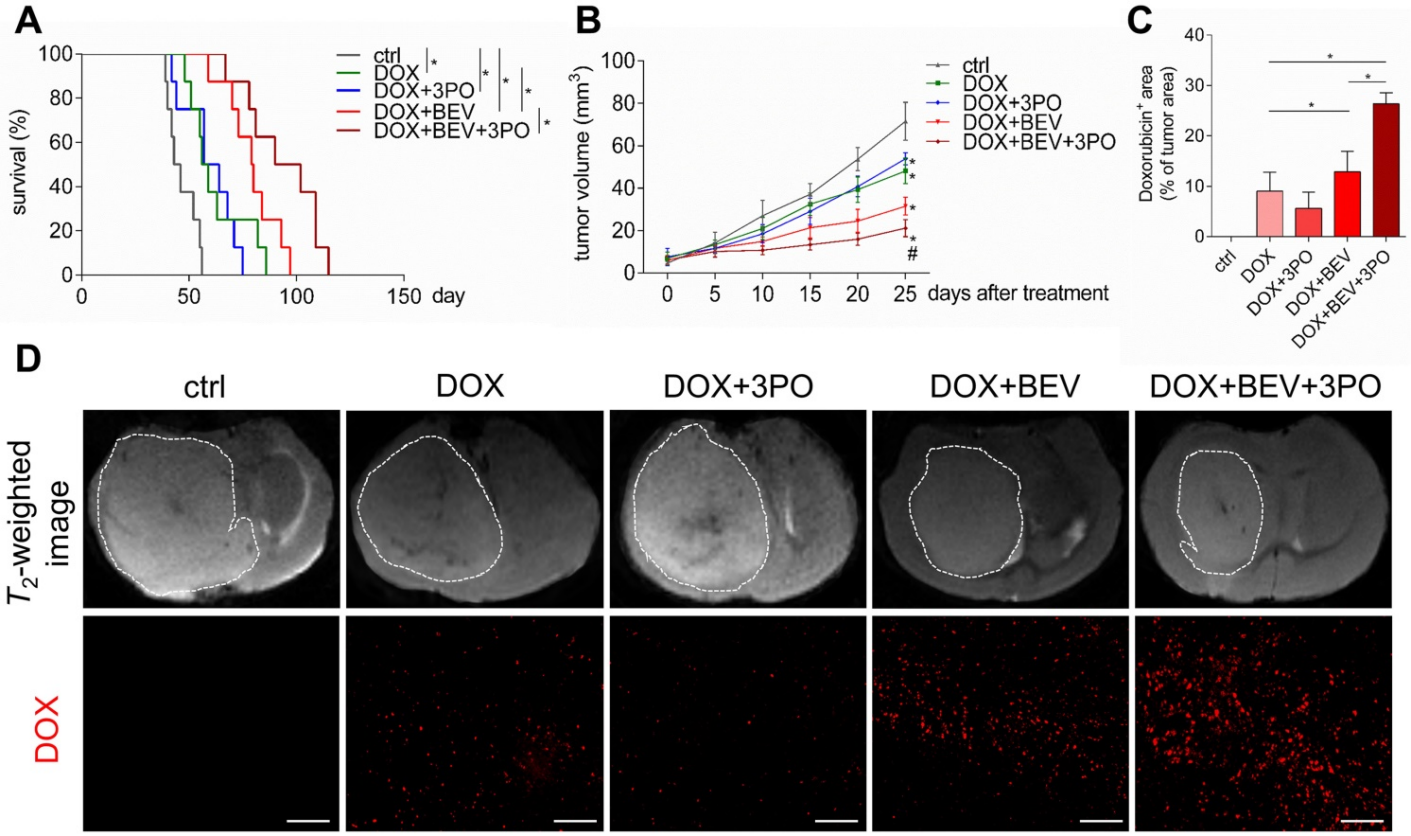
** Evaluation of doxorubicin therapeutic efficacy and its intratumoral distribution when combined with 3PO, BEV, or BEV+3PO treatments. (A)** Survivals of tumor-bearing mice from different therapy groups (*n* = 40). **(B)** Growths of GBM tumors in different therapy groups (*n* = 25). **(C)** Quantification of doxorubicin accumulation in tumor areas from different therapy groups (*n* = 25). **(D)** Representative *T*_2_-weighted images of tumor volumes and DOX-positive areas in tumor regions from different therapy groups. Scale bars, 50 µm. All data are means ± *SD*. ^*^P < 0.05, compared with control. ^#^*P* < 0.05, compared with the BEV group.

Given that TVN remodels the hostile tumor microenvironment into a more favorable circumstance characterized by improved oxygenation status, low acidity, and high drug delivery efficiency, thereby significantly improving chemotherapy efficacy 3,40,41; we next investigated whether a better outcome of chemotherapy gained from the sustained TVN by dual therapy. Consistent with previous studies 40,42,43, treatment with DOX and BEV significantly increased survival and delayed tumor growth compared with control or DOX monotherapy (Figures 5A-5B). Strikingly, these effects were more remarkable in the DOX+BEV+3PO triple therapy group (Figure 5B and 5D, and Table S1). Furthermore, we measured the drug delivery of DOX by detecting DOX accumulation in tumor regions. In line with the survival and tumor growth results, both BEV+DOX and triple therapy groups extended the DOX-positive area compared with DOX+3PO, DOX alone, or control groups. The accumulation of DOX in tumors treated with triple therapy was more significant than that seen in DOX+BEV group (Figures 5C-5D). These data suggest that BEV+3PO therapy further improves the effectiveness of DOX by enhancing drug delivery through a more durable TVN effect.

### Characterizing molecular profiling of dual therapy-induced TVN

**Figure 6:**
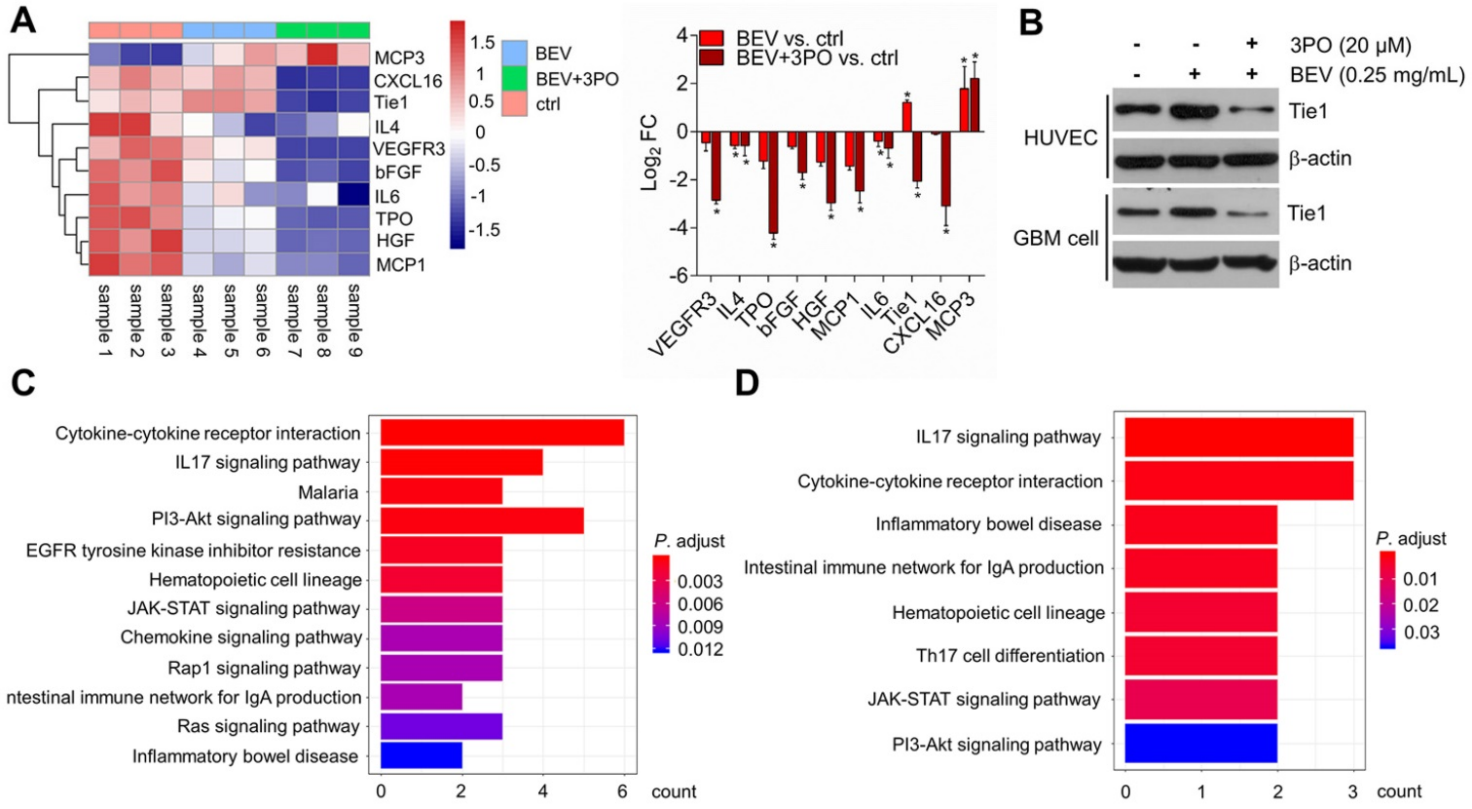
** Molecular profiling of angiogenesis-associated proteins after dual therapy and the effect of dual therapy on Tie1 expression. (A)** Heatmap and the fold change of the differently expressed proteins (*n* = 9). **(B)** Western blotting of Tie1 in ECs and tumor cells after treated with BEV or BEV+3PO for 24 h (*n* = 6). The top 12 relevant pathways based on DEPs from dual therapy group **(C)** and BEV monotherapy group **(D)**. All data are means ± *SD*. ^*^P < 0.05 compared with control.

To further investigate and understand how the synergistic effects of dual inhibition of VEGF and PFKFB3 improved the TVN in GBM, we performed protein microarray to characterize the molecular profiling of angiogenesis-related protein after BEV monotherapy or dual therapy. A total of 60 proteins were screened and 10 differentially expressed proteins (DEPs) were identified. Consistent with the previous study 44, both dual therapy and BEV monotherapy induced elevated MCP3 expression, suggesting therapeutic response. We found that only IL4 and IL6 were significantly downregulated in tumors treated with BEV monotherapy, indicating the limited efficacy after long-term use of VEGF blockade. In contrast, more proangiogenic cytokines including IL4 and IL6 were downregulated in dual therapy. Among the 10 DEPs, the Tie1 regulation was opposite between BEV and dual therapy, indicative of an important role of Tie1 in mediating TVN effect by dual therapy (Figure 6A). Tie1 is essential for vascular development and functionally involved in tumor angiogenesis independent on VEGF signaling 45-47. Notably, Tie1 is proven to be a key regulator in vascular stabilization and remodeling. Conditional deletion of Tie1 in ECs promotes TVN 48. This raises the question of whether dual therapy affects Tie1 expression in ECs as well as tumor cells. Western blotting further determined that dual therapy effectively inhibited Tie1 expression not only in ECs but in tumor cells compared with BEV monotherapy (Figure 6B), confirming that dual therapy downregulates the Tie1 expression. We further conducted pathway enrichment analysis to identify pathways significantly regulated by dual therapy. Several pathways such as cytokine-cytokine receptor interaction, PI3K/Akt, IL17, and JAK/STAT signaling pathways were downregulated in both BEV therapy and dual therapy with a more pronounced degree of enrichment in the latter (Figures 6C-6D and Tables S2-S3). Specifically, EGFR tyrosine kinase inhibitor resistance, chemokine, Ras, and Rap1 signaling pathways were downregulated exclusively in tumors treated with dual therapy, of which EGFR and Ras are known anti-normalizing pathways in TVN molecular regulation 3.

### Monitoring TVN process by DCE- and IVIM-MRI

**Figure 7:**
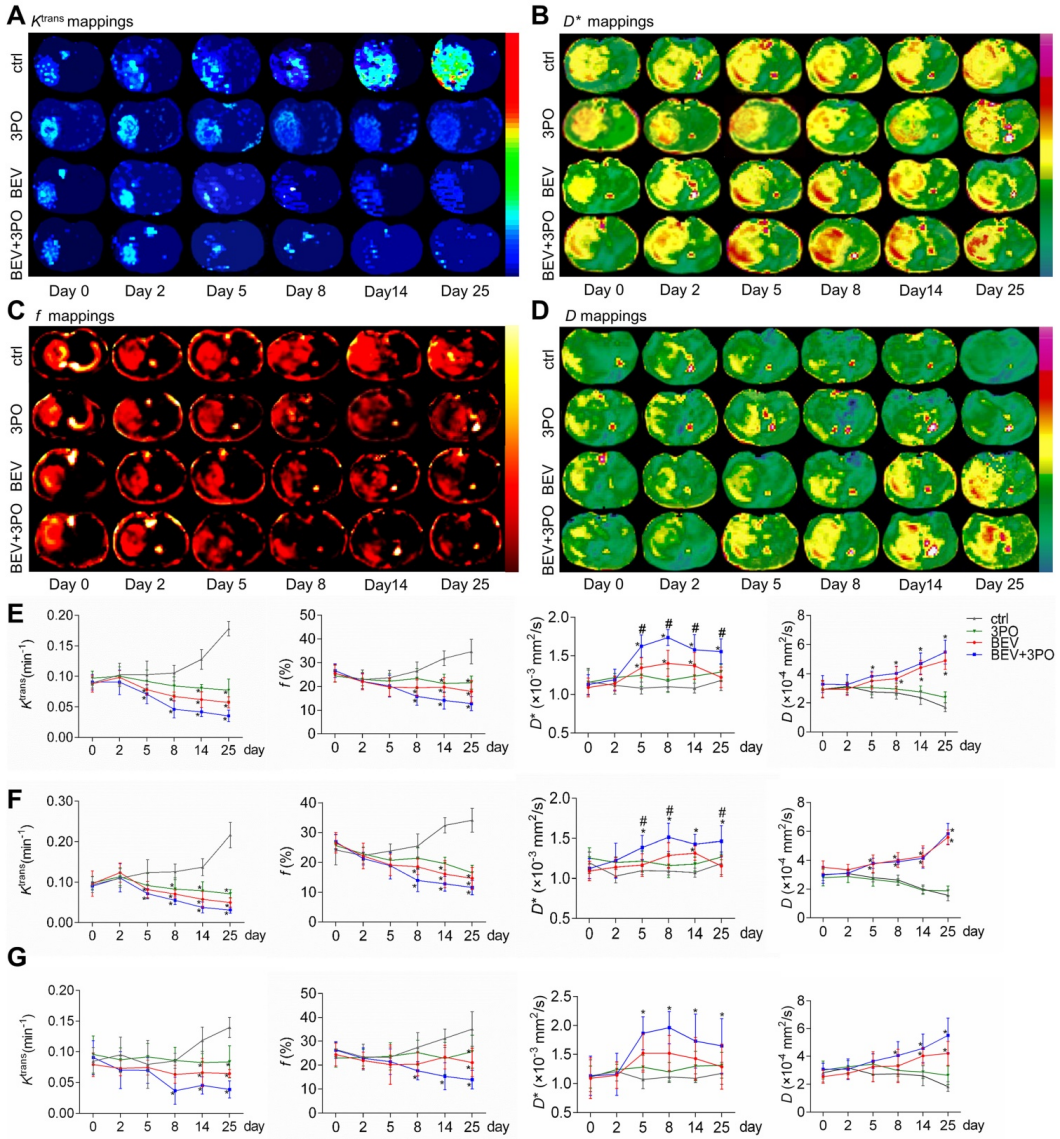
** Tumor vascular normalization monitored by DCE- and IVIM-MRI based on region-specific analysis.** Representative *K*^trans^ mappings **(A)**, D^*^ mappings **(B)**, *f* mappings **(C)**, and *D* mappings **(D)** at different time points from different treatment groups (*n* = 20). The longitudinal assessments of *K*^trans^, *f*, D^*^, and *D* in the whole tumor region **(E)**, tumor core **(F)**, and tumor rim **(G)**. All data are means ± *SD*. ^*^P < 0.05 compared with control. ^#^*P* < 0.05 compared with BEV group.

We performed DCE- and IVIM-MRI at sequential time points to monitor TVN process by BEV monotherapy or dual therapy. The downtrends of *K*^trans^ and *f* in the whole tumor region were observed in the BEV group and dual therapy group, indicating the decrease of vascular permeability and tumor angiogenesis (Figures 7A, 7C, and 7E). The D^*^ mappings in tumors treated with BEV therapy or dual therapy showed that D^*^ values were significantly increased at day 5, reaching a peak at day 8, suggesting the normalization of vascular function with increased blood flow as the previous study reported (Figure 7B) 49. Moreover, dual therapy increased D^*^ value to a greater extent than BEV monotherapy dose and maintained this effect by day 25 compared with control (Figure 7E). The *D* values in tumors treated with BEV or BEV+3PO were both increased gradually, suggesting the reduced tumor cellularity after treatments (Figure 7D). Due to that intrinsic intratumoral heterogeneity results in different therapeutic responses in tumor subregions, we further segmented the whole tumor region into two parts as tumor rim and tumor core to characterize the therapeutic responses in different tumor subregions. *K*^trans^, *f*, and *D* in tumor core and tumor rim were changed after BEV or BEV+3PO therapy with a greater extent in the dual therapy (Figures 7F-7G). However, we found the change of D^*^ value in BEV-treated tumors was relatively limited. Only D^*^ in the tumor core was increased at day 14 and no significant change was observed in the tumor rim. In contrast, both in tumor core and peripheral regions, D^*^ in the dual therapy-treated tumor has a statistical difference compared to control with a significant increment of D^*^ from day 5 to day 25. This result indicates that the addition of 3PO to BEV monotherapy induces a more homogenous and prominent TVN effect characterized by normalized vascular function not only in tumor core but in tumor periphery.

### Correlations between MRI parameters and TVN pathological indicators

**Figure 8:**
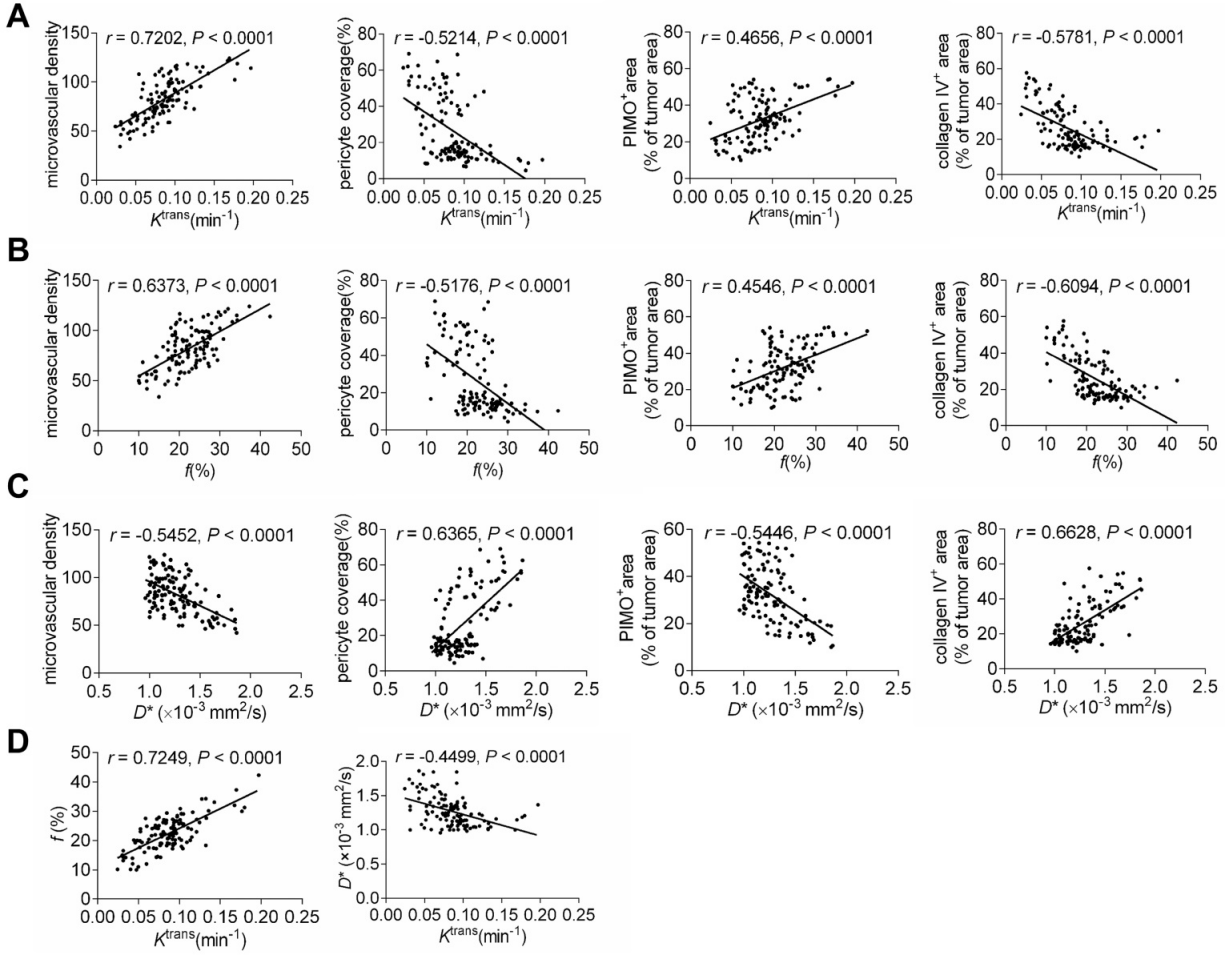
** Correlations between MRI parameters and histological indicators, as well as DCE- and IVIM-MRI parameters.** The correlations between *K*^trans^ and histological indicators **(A)**, *f* and histological indicators **(B)**, and D^*^ and histological indicators **(C)**. **(E)** The correlations between *K*^trans^ and *f* and *K*^trans^ and D^*^.

We further analyzed the correlations between perfusion-related MRI parameters and pathological indicators, as well as between IVIM and DCE-MRI parameters. As shown in Figures 8A-8C, *K*^trans^ and *f* were related to MVD intensively but had moderate correlations with PCI, collagen IV, and tumor hypoxia. In contrast, the correlations between D^*^ and normalization indicators were more intensive despite the moderate correlation with MVD. Correlation analysis of MRI parameters revealed that *K*^trans^ positively correlated with *f* but weakly with D^*^. This indicates that the IVIM parameter *f* is comparable to the DCE-MRI parameter *K*^trans^ to reflect histological characterizations post-treatment. Moreover, IVIM parameter D^*^ may be complementary to *K*^trans^ for monitoring the TVN window by evaluating pathological TVN features more precisely.

## Discussion

AAT initially aims to inhibit tumor vessel growth for starving tumors by cutting off the nutrition supply 50. However, long-term use of AAT facilitates the formation of a hostile tumor microenvironment characterized by hypoxia and acidity, thus promoting tumor progression and malignant transformation, and deteriorating hypoxia again to generate a vicious cycle 10,51. In contrast to traditional AAT, normalizing tumor vasculature with low-dose VEGF blockade is an alternative promising approach that remodels a favorable tumor microenvironment for optimally combining other anticancer therapies to synergize effectiveness 7,52. However, the TVN effect is transient due to AAT resistance 3,9. An urgent need exists for sustaining the TVN effect by interfering with new potential targets besides the VEGF axis. We here first reported a novel combined strategy by dual inhibition of VEGF and PFKFB3 to sustain TVN effect, thereby alleviating tumor hypoxia, reducing lactate production, and improving chemotherapy efficacy. Moreover, IVIM-MRI parameter D^*^ shows better correlations with pathological TVN indicators than traditional DCE-MRI parameter *K*^trans^ thus may be a candidate biomarker complementary to* K*^trans^ for monitoring the TVN process.

Having established that anaerobic glycolysis mediates AAT resistance in GBM and plays a critical role in tumor angiogenesis 40,53. We hypothesized that AAT efficacy by BEV could improve by inhibiting the glycolytic activator PFKFB3. As expected, our data demonstrated that PFKFB3 is significantly increased after BEV monotherapy despite delayed tumor growth and increased survival. In contrast, the addition of 3PO reduces the expression of BEV-induced PFKFB3 and greatly lowers tumor growth and extends survival. However, it is worth noting that 3PO monotherapy may not interfere with the growth of GBM cells and has little effect on tumor vessels (Figures 2 and 3). This may partially be explained by the low dose of 3PO used in this study and the greater dependence of VEGF in GBM vasculature. A low dose (25 mg/kg) of 3PO is insufficient to antitumor effect. This dosage, however, is sufficient to induce TVN leading to a reduction in cancer cell invasion, metastasis, and tumor hypoxia 53. In contrast, the high dose (70 mg/kg) of 3PO reduces cancer cell proliferation but fails to improve vessel maturation, perfusion, and tumor oxygenation, thereby rendering tumor vessels leakier and facilitating cancer cell dissemination 54. Also, GBM neovascularization is mainly mediated by VEGF signaling but can shift rapidly into other alternative mechanisms such as anaerobic glycolysis to recruit vasculature after VEGF blockade 10,13,55. Therefore, these data suggest that PFKFB3 may be more relevant as a potential target to improve the therapeutic efficacy in GBM under the context of VEGF pathway inhibition.

Previous studies have demonstrated that TVN leads to better prognosis in preclinical models and GBM patients 4-6. Indeed, our results showed that dual therapy enhances the TVN effect with a durable TVN window and a greater normalized extent than BEV monotherapy dose, resulting in more extended survival in PDX GBM models. Hypoxia is the central indicator of vascular normalization 3. Increased oxygenation status remodels a favorable tumor microenvironment sensitive to chemotherapy 38,56. Paralleled with the resulting extension of vascular normalization, dual therapy alleviates tumor hypoxia maintaining at a low level by day 25 post-treatment, which is much longer than that in the BEV monotherapy group. We also observed that dual therapy reduces the production of BEV-induced lactate and LDHA expression, remodeling a less acidic tumor microenvironment. TVN offers a window of opportunity for combined other antitumor therapies. The better outcome of chemotherapy is obtained when therapy administrated during the TVN window 3,57,58. To investigate whether dual therapy could improve the chemotherapy outcome by sustained TVN effect, we used the chemotherapeutic agent DOX. Consistent with previous studies 40,42,43, our data showed that BEV-induced TVN enhances the effectiveness of DOX therapy with significantly increased survival and delayed tumor growth. The accumulation of DOX in BEV-treated tumors was increased significantly compared with DOX therapy alone or control. Notably, these effects were even more enhanced in DOX+BEV+3PO triple therapy. We also observed that the error bar of DOX-positive areas in triple therapy is smaller than that in DOX+BEV dual therapy despite no statistical difference (Figure 5C), indicating that the combination of BEV and 3PO induces a more homogenous intratumoral distribution of DOX. In summary, our finding demonstrated that dual therapy sustains the TVN effect, thereby alleviating tumor hypoxia, reducing lactate production, and enhancing drug delivery more significantly compared with BEV monotherapy.

Since that dual therapy induces a durable TVN effect *in vivo*, we performed the angiogenesis-associated protein array to characterize molecular profiling of the TVN achieved by dual therapy. 10 DEPs were identified uncovering the treatment-specific protein expression profiling. The downregulated proteins in dual therapy are more than that in BEV monotherapy, indicative of the wider range of effect on several angiogenic proteins after combining BEV with 3PO. Moreover, we found that Tie1 is the most significant DEP in the comparison between BEV and dual therapy groups. Given the known and important role of Tie1 in TVN and the significant difference of Tie1 expression between BEV and BEV+3PO groups 48, we speculated that Tie1 may play an important role in dual therapy-induced TVN. In line with the sustained TVN effect at day 25, our findings demonstrated that both Tie1 expressions in ECs and GBM cells were significantly reduced in tumors treated with dual therapy. This result is consistent with the previous study reporting that conditional deletion of Tie1 in ECs normalized tumor vessels in Lewis lung cancer and melanoma models 48. Based on this point of view, our findings suggest that dual therapy likely reinforces the TVN effect by inhibiting Tie1 expression in ECs and tumor cells. Undoubtedly, the molecular mechanism of TVN is sophisticated resulting from the regulation of several angiogenic cytokines and signaling pathways 3. Our data do not exclude the possibility that dual therapy affects TVN by regulating other molecules or by affecting other stroma cells. Still, considering that Tie1 has been proven in regulating TVN and the impact of dual therapy on Tie1 expression, Tie1 is at least one of the important factors in mediating TVN by dual therapy in GBM. The bioinformatics analysis further revealed that dual therapy downregulates several signaling pathways exclusively, of which EGFR and Ras are anti-normalizing pathways. Indeed, the limitation of the current study is that we do not fully clarify the regulation of Tie1 in TVN by dual therapy. The further study focused on the Tie1 effect on the regulation of dual therapy-induced TVN is warranted. Collectively, the results from the current study provide molecular insight into the synergistic effect of BEV and 3PO on TVN and may serve as a preliminary resource for clinical translation when combined with the human database.

Monitoring TVN process is of great clinical significance to determine TVN window, thereby judging treatment decision when combined with other anticancer therapies. The DCE-MRI parameter *K*^trans^ is recommended as the standard endpoint of AAT according to QIBA. However, DCE-MRI is limited by the GBCAs deposition when used frequently for determining the TVN window. Currently, IVIM-MRI is entering the clinical field to evaluate tissue perfusion without GBCAs administration, and the ability to analyze non-Gaussian diffusion is more sensitive to tissue features 26. We here performed DCE- and IVIM-MRI to monitor TVN window. Meanwhile, we analyzed the correlations between MRI parameters and TVN histological indicators. The DCE-MRI parameter *K*^trans^ and IVIM-MRI parameter *f* are decreased after BEV or BEV+3PO therapies, indicative of reduced tumor angiogenesis. Indeed, and consistent with previous studies 49,59,60, these two parameters are positively correlated with tumor MVD. IVIM parameter D^*^ is associated with blood flow 26,27. Consistent with the previous study reporting that increased D^*^ value reflects the restoration of vascular function 49, D^*^ is significantly increased during the TVN window. Notably, the increments of D^*^ in tumor core and tumor rim are both elevated in the dual therapy group to a greater extent compared with the BEV monotherapy group. However, no statistically significant difference of D^*^ is observed in tumor rim between the BEV treatment group and control. Also, we found that in tumor rim, the relative changes of *K*^trans^ and *f* to control in dual therapy are slightly greater than these in BEV monotherapy despite no statistical difference. These findings were consistent with the result of drug delivery showing a more homogenous distribution of DOX in dual therapy-treated tumors. The high heterogeneity of TVN effect in tumors treated with BEV monotherapy reflected by DCE- and IVIM-MRI may partially arise from vessel co-option. Vessel co-option is a typical process in GBM periphery characterized by tumor cells cuffing around normal vessels, mediating intrinsic and acquired resistance to AAT 61-63. Due to the cuff structure formed by the compact arrangement of tumor cells and the limited efficacy of BEV, it is likely that a fraction of vessels in the tumor rim may be still compressed after BEV monotherapy and thus the finite change of blood flow evaluated by D^*^. Still, it is worth verifying and elucidating the relationship between the heterogeneous of BEV-induced TVN and vessel co-option in GBM in the future.

The correlations between perfusion-related MRI parameters and TVN indicators were further analyzed. We showed that all perfusion-related parameters were significantly related to vascular pathological indicators and tumor hypoxia. Notably, The correlations between D^*^ and TVN indicators (PCI, collagen IV, hypoxia) are more intensive than between *K*^trans^ and corresponding indicators. *K*^trans^ is a quantitative parameter to evaluate vascular function determined by tissue uptake or removal of GBCAs as they pass through the vasculature 64. Both vascular permeability and blood volume contribute to the measurement of *K*^trans^ 65. Hence, the *K*^trans^ value is a result to comprehensively evaluate the vascular function and influenced by some factors including permeability, blood flow, extravascular extracellular space, and interstitial fluid pressure 65,66. In contrast, D^*^ is more specific to *K*^trans^ which is thought to be a pure indicator evaluating the mean transit time of cerebral blood flow 27. Increased D^*^ is indicative of increased flow velocity 27,49. Therefore, considering the mixture implication and several impact factors of *K*^trans^, it is expected that D^*^correlates with TVN pathological indicators with stronger relevancy than *K*^trans^ dose. Further studies are needed that using a more complicated pharmacokinetic model to separate mixed factors such as vascular permeability and blood flow, thereby drawing a more comprehensive conclusion in the relationship between other DCE-MRI parameters and TVN indicators.

In conclusion, our findings provide direct evidence that dual blockade of VEGF and PFKFB3 reinforces the TVN effect in GBM. The sustained TVN alleviates tumor hypoxia, reduces LDHA and lactate productions, and creates a sustained window of opportunity to improve chemotherapy efficacy. In contrast to traditional TVN strategy by AAT, we propose a new strategy to fortify the TVN effect, which may be an alternative to fill gaps in current TVN strategies and provide preliminary enlightenment to design a versatile nanoparticle co-targeting VEGF and PFKF3 for normalizing tumor microenvironment persistently. Additionally, our findings suggest that IVIM parameter D^*^ is strongly related to TVN histological indicators and may compensate *K*^trans^ as a complementary imaging biomarker to monitor TVN without GBCAs injection. This provides a new resource of the clinically translatable imaging biomarkers for guidance on treatment decisions during TVN window.

## Supplementary Material

Supplementary figures and tables.

## Abbreviations

AAT: anti-angiogenic therapy
BEV: bevacizumab
Cho: choline
Cr: creatine
DCE: dynamic contrast-enhanced
DOX: doxorubicin
DEPs: differentially expressed proteins
ECs: endothelial cells
GBCAs: Gd-based contrast agents
TVN: tumor vascular normalization
GBM: glioblastoma
HUVECs: human umbilical vein endothelial cells
IVIM: intravoxel incoherent motion
LDHA: lactate dehydrogenase-A
Lac: lactate
Lip: lipid
MVD: microvascular density
MRI: magnetic resonance imaging
MRS: magnetic resonance spectroscopy
NAA: N-acetyl aspartic acid
PCI: pericyte coverage index
PDX: patient-derived xenograft
PFKFB3: 6-phosphofructo-2-kinase/fructose-2, 6-bisphosphatase-3
PIMO: pimonidazole
QIBA: Quantitative Imaging Biomarkers Alliance
TBST: TBS-Tween20
VEGF: vascular endothelial growth factor
TE: echo time
TR: repetition time
αSMA: α-smooth muscle actin
3PO: 3-(3-Pyridinyl)-1-(4-pyridinyl)-2-propen-1-one
VEGF: vascular endothelial growth factor
DAKO: 3, 3^'^-diaminobenzidine
