# Dual inhibition of PFKFB3 and VEGF normalizes tumor vasculature, reduces lactate production, and improves chemotherapy in glioblastoma: insights from protein expression profiling and MRI: Erratum

**DOI:** 10.7150/thno.92042

**Published:** 2023-11-17

**Authors:** Junfeng Zhang, Wei Xue, Kai Xu, Liang Yi, Yu Guo, Tian Xie, Haipeng Tong, Bo Zhou, Shunan Wang, Qing Li, Heng Liu, Xiao Chen, Jingqin Fang, Weiguo Zhang

**Affiliations:** 1 Department of Radiology, Daping Hospital, Army Medical University, Chongqing, 400042, China.; 2 Department of Neurosurgery, Daping Hospital, Army Medical University, Chongqing, 400042, China.; 3 Department of Oncology, Daping Hospital, Army Medical University, Chongqing, 400042, China.; 4 Department of Radiology, PLA Rocket Force Characteristic Medical Center, Beijing, 100088, China.; 5 Department of Nuclear Medicine, Daping Hospital, Army Medical University, Chongqing, 400042, China.; 6 Chongqing Clinical Research Center of Imaging and Nuclear Medicine, Chongqing, 400042, China.

The authors regret that some inappropriate images were contained in Figure 3 from our paper. The immunostaining images of CD31/αSMA, PIMO, collagen IV and LDHA at day 0, day 2, day 14 and day 25 from BEV+3PO, BEV and control groups were mis-pasted when choosing representative images from the large image data for typesetting. The correct version of Figure 3 appears below and these corrections made in this erratum do not affect the original data and conclusions. The authors apologize for any inconvenience that the errors may have caused.

## Figures and Tables

**Figure 3:**
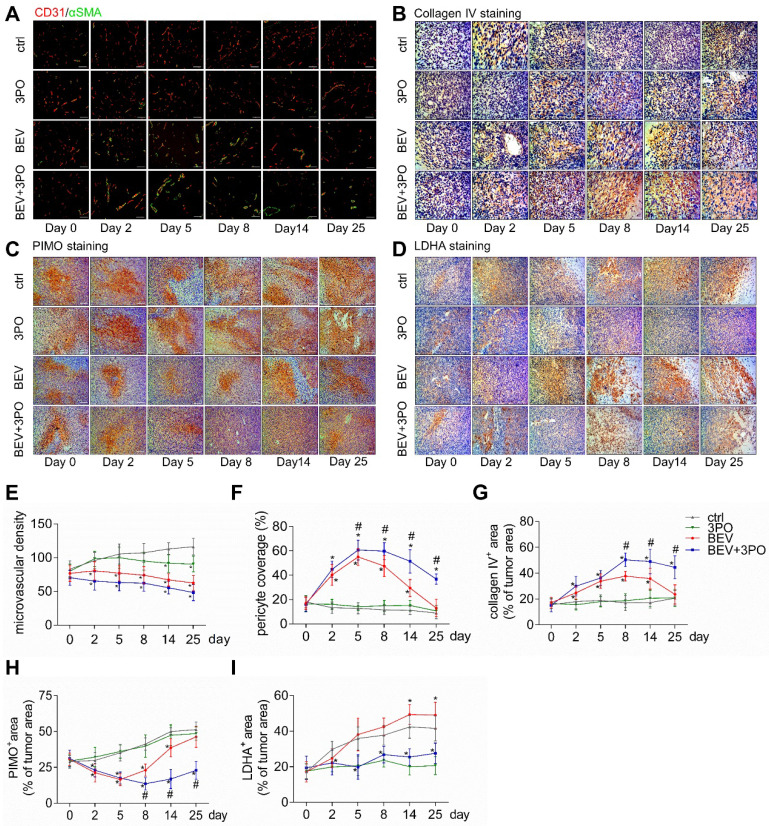
** Histological analysis of tumors at different time points after treatments.** Representative images of double CD31 and αSMA-stained **(A)**, collagenⅣ-stained **(B),** PIMO-stained **(C)**, and LDHA-stained **(D)** GBM sections at different time points from different treatment groups (*n* = 120). The longitudinal assessments of MVD **(E)**, pericyte coverage **(F)**, collagenⅣexpression **(G)**, tumor hypoxia **(H)**, and LDHA expression **(I)** in the control, 3PO, BEV, and BEV+3PO groups at different time points. Scale bars, 50 μm **(A, B)**, 100 μm **(C, D)**. All data are means ± *SD*. ^*^P < 0.05, compared with control. ^#^*P* < 0.05, compared with the BEV group.

