# Supplementary material for: Dual inhibition of PFKFB3 and VEGF normalizes tumor vasculature, reduces lactate production, and improves chemotherapy in glioblastoma: insights from protein expression profiling and MRI
Source: Theranostics. 2020 Jun 5;10(16):7245–59. doi: 10.7150/thno.44427 (PMC7330843; doi:10.7150/thno.44427)
Supplement: Supplementary file 1 — Supplementary figures and tables. [file thnov10p7245s1.pdf]

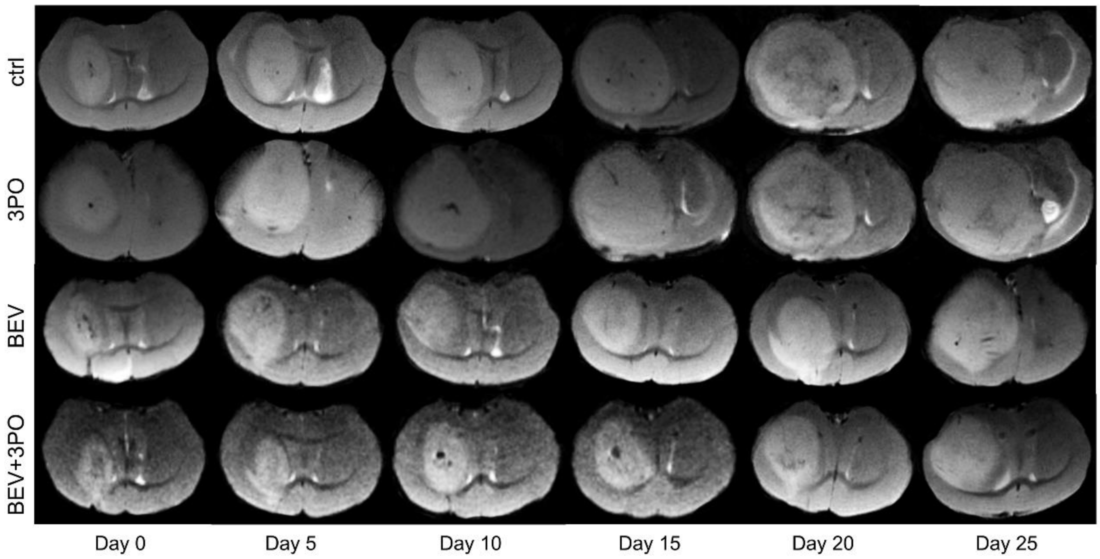

Figure S1. Representative T_2-weighted images of tumor volumes at different time points from different therapy groups.

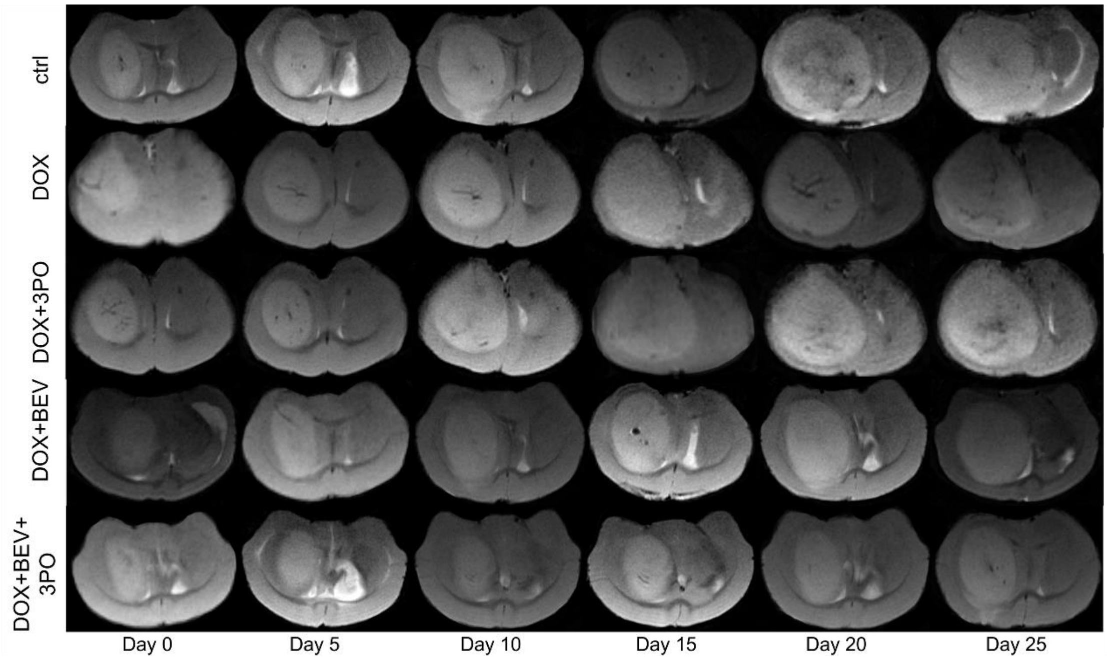

Figure S2. Representative T_2-weighted images of tumor volumes at different time points from different therapy groups.

Table. S1 Median survival of different treatment groups

Group	Median survival
Control	44.5
3PO	48
BEV	68.5
BEV+3PO	81
DOX	57.5
DOX+3PO	61
BEV+DOX	80
BEV+3PO+DOX	95

Table. S2 Pathway analysis of the DEPs from BEV monotherapy group

**Description**	**Gene ID**	**Count**	**Adjusted *P* value**
IL-17 signaling pathway	IL4/IL6/MCP3	3	<0.0001
Cytokine-cytokine receptor interaction	IL4/IL6/MCP3	3	<0.01
Intestinal immune network for IgA production	IL4/IL6	2	<0.01
Inflammatory bowel disease	IL4/IL6	2	<0.01
Hematopoietic cell lineage	IL4/IL6	2	<0.01
Th17 cell differentiation	IL4/IL6	2	<0.01
JAK-STAT signaling pathway	IL4/IL6	2	<0.01
PI3K-Akt signaling pathway	IL4/IL6	2	<0.05

Fisher's exact test was used for pathway enrichment analysis. A significant adjusted threshold of $P < 0.05$ was used.

Table. S3 Pathway analysis of the DEPs from BEV+3PO therapy group

Description	Gene ID	Count	Adjusted *P* value
Cytokine-cytokine receptor interaction	IL4/IL6/MCP3	6	<0.0001
IL-17 signaling pathway	IL4/IL6/MCP3	4	<0.0001
Malaria	IL4/IL6	3	<0.001
PI3K-Akt signaling pathway	IL4/IL6	5	<0.001
EGFR tyrosine kinase inhibitor resistance	IL4/IL6	3	<0.001
Hematopoietic cell lineage	IL4/IL6	3	<0.01
JAK-STAT signaling pathway	IL4/IL6	3	<0.01
Chemokine signaling pathway	IL4/IL6	3	<0.01
Rap1 signaling pathway	VEGFR3/FGF2/HGF	2	<0.01
Intestinal immune network for IgA production	VEGFR3/FGF2/HGF	3	<0.01
Ras signaling pathway	IL4/IL6	3	<0.05
Inflammatory bowel disease	FGF2/HGF	2	<0.05
Melanoma	FLT4/FGF2/HGF	2	<0.05
MAPK signaling pathway	IL6/MCP1	3	<0.05
Rheumatoid arthritis	IL6/MCP1	2	<0.05
AGE-RAGE signaling pathway in diabetic complications	IL6/MCP1	2	<0.05
Chagas disease (American trypanosomiasis)	IL4/IL6	2	<0.05
Th17 cell differentiation	IL6/MCP1	2	<0.05
TNF signaling pathway	FLT4/FGF2	2	<0.05
Breast cancer	FGF2/HGF	2	<0.05
Gastric cancer	IL6/MCP1	2	<0.05
Influenza A	IL6/MCP1	2	<0.05

Fisher's exact test was used for pathway enrichment analysis. A significant adjusted threshold of $P < 0.05$ was used.
